# Rate of Intracranial Hemorrhage After Minor Head Injury

**DOI:** 10.7759/cureus.10653

**Published:** 2020-09-25

**Authors:** Phillip A Bonney, Amy Briggs, Robert G Briggs, Casey A Jarvis, Frank Attenello, Steven L Giannotta

**Affiliations:** 1 Department of Neurosurgery, University of Oklahoma Health Sciences Center, Norman, USA; 2 Department of Neurosurgery, University of Southern California Keck School of Medicine, Los Angeles, USA; 3 Emergency Medicine, University of Southern California Keck School of Medicine, Los Angeles, USA

**Keywords:** blunt head trauma, clinical decision rule, emergency room, head ct, intracranial hemorrhage, mild traumatic brain injury, subdural hematoma, subarachnoid hemorrhage, hemorrhagic contusion, intracerebral hematoma

## Abstract

Introduction: Computed tomography scans of the head (CTH) are an important component of the initial patient evaluation after blunt head trauma in select patients. Here we review findings of CTH performed for mild traumatic brain injury (TBI) at a Level I trauma center over a two-year period. We subsequently discuss the role and limitations of published clinical decision rules aiming to decrease unnecessary CTH in mild TBI patients.

Methods: We reviewed all Emergency Department CTH obtained after blunt head trauma between 2010 and 2011. Patient demographics and radiology report texts were collected. Reports were cross-referenced with our institutional trauma database to obtain initial Glasgow Coma Scale (GCS). Mild TBI was defined by an initial GCS 13-15 with or without loss of consciousness or post-traumatic amnesia.

Results: There were 5,634 mild TBI patients evaluated with CTH. A total of 477 scans (8.5%) were positive for intracranial hemorrhage. Of these, 188 (39.4%) showed more than one type of intracranial hemorrhage. The most common findings were subdural hematomas (262, 4.7% of scans), traumatic subarachnoid hemorrhages (252, 4.5% of scans), and cerebral contusions/intraparenchymal hematomas (212, 3.8% of scans). Older age (p<0.001) and male gender (p<0.001) were associated with positive CTH.

Conclusions: The rate of positive CTH in mild TBI patients in our population falls within a historical range. The clinical and medicolegal implications of missed intracranial hemorrhage have remained important factors limiting the implementation of clinical decision rules in screening mild TBI patients for CTH.

## Introduction

The initial evaluation of patients sustaining traumatic head injuries commonly includes a head computed tomography scan (CTH). In mild traumatic brain injury (TBI) patients, who comprise the majority of TBI, attention has been given to stratifying those who should and should not be evaluated with CTH. In most studies, traumatic intracranial findings are present in 5-10% of mild TBI patients who receive CTH, and neurosurgical intervention is necessary in 0.5-1% [1-4]. However, mild TBI is a nebulous entity, variably defined by Glasgow Coma Scale (GCS) 13-15, GCS 14-15, or GCS 15 and occurring with or without loss of consciousness or retrograde amnesia [5,6], which has confounded its study in the context of diagnostic evaluation.

Multiple prospective observational studies have been used to establish sets of clinical decision rules to identify patients who do not warrant testing with CTH. The premise of such tools is to reduce the unnecessary use of CTH in patients unlikely to harbor traumatic findings, while identifying patients at risk for intracranial hemorrhage. Though several observational studies have assessed the value of such tools at potentially reducing unnecessary CTH [2], it is not clear that such efforts have led to reduction in CTH usage. Rather, data suggest that CTH use in emergency departments is increasing [7,8].

Commonly cited reasons to limit CT utilization after mild TBI include radiation exposure and cost. While these are important considerations, they should not be overstated, as the radiation exposure from a single CTH is considerably less than that of most other CT scans. The number of new cancer diagnoses attributable to a single CTH has been estimated at one in 4,000-20,000 patients for those five years of age or older [9]. Avoiding needless CTH reduces costs [10], but if clinically significant hemorrhages are missed, costs may instead be increased [11]. Further, the clinical and medicolegal implications of an unrecognized intracranial hematoma have remained important drivers of practice patterns amongst emergency medicine providers [12]. This latter consideration likely accounts for increasing CTH usage, as no decision instrument has demonstrated 100% sensitivity across populations tested.

Our purpose in this study was to evaluate the incidence of intracranial hemorrhage in patients with mild traumatic brain injury (TBI) at our institution, an urban, 600-bed Level I trauma center. To do so, we performed a retrospective review of CTHs obtained in the Emergency Department for blunt head trauma over a two-year period. We discuss our findings in the context of prior literature.

## Materials and methods

We identified all head CTs (CTHs) obtained in the setting of trauma from the Emergency Department of LAC+USC Medical Center, a 600-bed Level I Trauma Center in East Los Angeles, between January 1, 2010 and December 31, 2011. Data for the study were obtained using Montage, a database of radiology reports that can be queried by any text present within a report. We searched scans for any of the following keywords: trauma, blunt, motorcycle, motor vehicle, car, accident, fall, MVC, MCC, collision, pedestrian. 

For all scans meeting the above criteria, patient age, patient gender, scan date, scan time, and radiology report text were exported into Excel® (Microsoft, Redmond, WA, USA). Duplicate studies were removed. Follow-up studies were removed. Indications for scans were reviewed to confirm the indication was blunt head trauma. Reports were then reviewed for presence of any of the following: subdural hematoma, subarachnoid hemorrhage, intraparenchymal/intracerebral hematoma, hemorrhagic contusion, or intraventricular hematoma. Reports that included any of these keywords were considered for inclusion and reviewed in detail. 

A CTH was considered positive if the radiology report documented an intracranial hemorrhage. In cases in which the read was not definitive (e.g. “possible” or “probable”), the scan was considered positive. Findings not related to acute blunt head trauma, including penetrating trauma and chronic pathology, were excluded. Acute non-traumatic intracranial hemorrhages were considered positive if the patient presented as a potential traumatic mechanism. For small hemorrhages in which the type of hemorrhage could not be clearly established (e.g. extra-axial hematoma representing possible subdural or epidural blood products), a single compartment was assigned in alternating fashion. Linear, basilar, and depressed skull fractures did not constitute positive findings unless intracranial hemorrhage was also present. Patients with delayed presentations after head injury were included in the study. 

The LAC+USC Trauma Registry was cross-referenced over the same time period to obtain presenting GCS data. Mild TBI was defined as patients presenting with GCS 13-15 with or without loss of consciousness or post-traumatic amnesia. Patients with minor injuries are not typically included in the Trauma Registry, and as such, only a fraction of the Montage patient cohort was represented in the Trauma Registry. For this reason, the mild TBI cohort comprised two groups: (1) patients in the trauma registry with GCS 13-15 and (2) patients not represented in the trauma registry, except those with positive CTH in which chart review revealed a GCS less than 13. 

The paradigm for screening blunt head trauma patients for CTH at our institution is not protocolized. While published tools including the Canadian CT Head Rule (CCHR) [13] and the New Orleans Criteria (NOC) [1] are commonly referenced, their use is on a per-provider basis, and the decision to obtain CTH is at the discretion of the Emergency Department physician treating team. 

Descriptive statistics were used to summarize demographic data. Two-sided student t-test was used to compare continuous data. Chi-square test was used to compare categorical data. A p-value <0.05 was considered significant. Statistics were performed using Statistical Product and Service Solutions (SPSS®) version 25 (IBM Corp., Armonk, NY, USA) 

## Results

Positive CTH in mild TBI patients

Over the two-year study period, our search identified 5,634 mild TBI patients. The median age of patients was 45 years (interquartile range 30 - 58 years). Patient age was bimodally distributed, with peaks in the third and sixth decades. The male-to-female ratio was 2.6:1.

In CTH for mild TBI patients, intracranial hemorrhage was present in 477 (8.5%). Findings are listed in Table 1. Among positive CTH, 188 (39.4%) showed more than one type of intracranial hemorrhage. Findings consisted of 262 acute subdural hematomas (4.7% of scans), 252 traumatic subarachnoid hemorrhages (4.5% of scans), 212 hemorrhagic contusions or intraparenchymal hematomas (3.8% of scans), 45 epidural hematomas (0.8% of scans), and 20 intraventricular hematomas (0.4% of scans). 

**Table 1:** Rates of Intracranial Hemorrhage in Patients with Mild TBI

Characteristic	N (%)
Presence of Intracranial Hemorrhage	477 (8.5%)
Type of Intracranial Hemorrhage	
Subdural hematoma	262 (4.7%)
Subarachnoid hemorrhage	252 (4.5%)
Hemorrhagic contusion/intraparenchymal hematoma	212 (3.8%)
Epidural hematoma	45 (0.8%)
Intraventricular hematoma	20 (0.4%)
Number of Intracranial Hemorrhages	
One	289 (60.2%)
Two or more	188 (39.8%)

**Figure 1:**
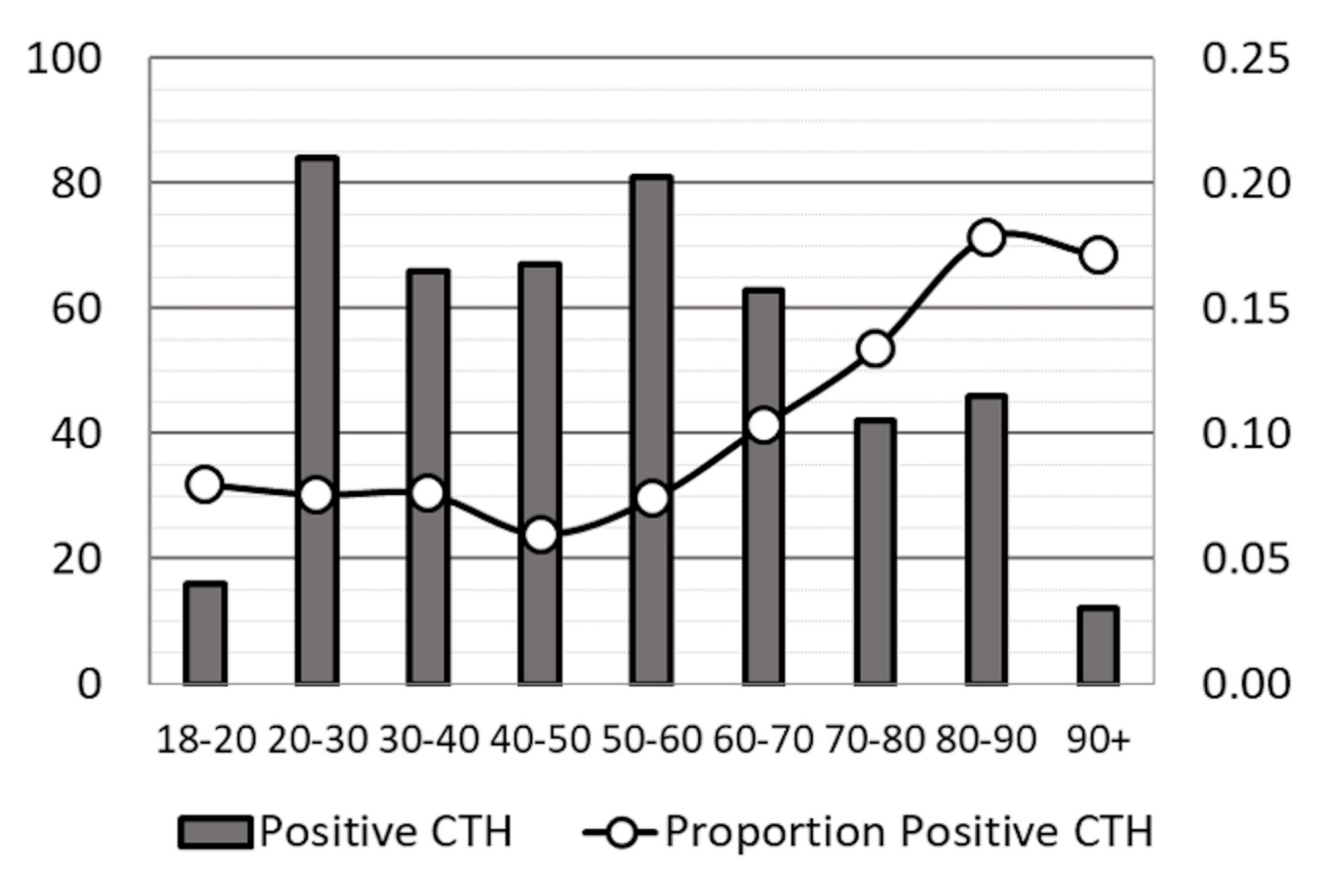
Number of positive CTH by decade (dark bars, left y-axis) and proportion of positive CTH (white circles, right y-axis).

The number and rate of positive CTH by decade of life are displayed in Figure 1 and Table 2. The rate of hemorrhage with increasing decade age displayed a slight downward trend between the second and fifth decades, from 8% to 6%. From the fifth decade onward, a positive linear relationship was evident, increasing at a rate of approximately 2-4% per decade, up to 18% by the ninth decade. A higher rate of positive CTH was seen in male patients (9.7% vs. 7.1%, p=0.002). A higher rate of positive CTH was found in mild TBI trauma registry patients than in non-trauma registry patients (13.0% vs. 2.8%, p=0.0001).

**Table 2:** Effect of Patient Gender and Age on Positive CTH for Mild TBI

Characteristic	Positive CTH/Total	p-value
Gender		
Female	103/1576 (6.5%)	0.001
Male	374/4058 (9.2%)	
Age Group		
18-20	16/200 (8.0%)	<0.001
20-30	84/1106 (7.6%)	
30-40	66/867 (7.6%)	
40-50	67/1116 (6.0%)	
50-60	81/1094 (7.4%)	
60-70	63/609 (10.3%)	
70-80	42/314 (13.4%)	
80-90	46/258 (17.8%)	
90+	12/70 (17.1%)	

## Discussion

In the present study, we sought to assess the incidence of acute intracranial hemorrhage in head CTs (CTH) obtained in a mild traumatic brain injury (TBI) population in a high-volume American trauma center. Our primary analysis revealed a positive CTH rate of 8.5%. Forty-four percent of positive CTH showed more than one type of intracranial hemorrhage. The most common hemorrhages were subdural hematomas (present in 4.7% of patients), subarachnoid hemorrhages (4.5%), and hemorrhage contusions/intracerebral hematomas (3.8%). Older age and male gender were associated with positive CTH findings. 

History of CTH for TBI

In early usage in the 1970s and 1980s, CTH after head trauma was primarily centered around severe TBI [14]. Consensus opinion maintained that patients with little-to-no signs or symptoms warranted no imaging, and those with moderate risk - defined by many criteria qualifying as high risk in more recent decision tools - be observed and only receive CTH or skull x-ray if deterioration occurred [15]. Several subsequent reports detailed high rates of intracranial hemorrhage in patients with benign neurologic exams, however, and recommended CTH for all patients with loss of consciousness or post-traumatic amnesia [16,17]. In these reports, rates of abnormal findings in such patients were approximately 18-22%, though these rates include non-hemorrhage pathologies such as edema and certain skull fractures [16,17]. The rates of neurosurgical intervention ranged from 4% to 5%. Even studies with lower rates of positive findings recommended CTH in most GCS 15 patients due to the need for neurosurgical intervention in a small number [18]. 

Later work focused on better identifying patients at risk for positive CTH findings [19-21]. Predictors of intracranial hemorrhage in these studies included older age, signs of skull fracture, neurologic deficit, ethanol intoxication, post-traumatic amnesia, headache, nausea, vomiting, certain mechanisms of injury, loss of consciousness, shunted hydrocephalus, GCS <15, coagulopathy, and extracranial injury [19-22].

Clinical decision tools for mild TBI 

Several decision tools were subsequently developed to help guide the use of CTH in mild TBI (Table 3). The New Orleans Criteria (NOC) were developed from a prospective analysis of 520 patients with GCS 15 and loss of consciousness or amnesia [1]. A notable aspect of NOC is the requirement to obtain CTH for any patient with evidence of injury above the clavicle, which partially explains the low specificity of this tool. The American College of Emergency Physicians subsequently issued a Class A recommendation that CTH is not indicated for patients not meeting NOC [23]. 

**Table 3:** Summary of North American Clinical Decision Rules for Obtaining CTH after Blunt Head Trauma CTH: computed tomography of the head, GCS: Glasgow Coma Scale

Canadian CT Head Rule (CCHR)	New Orleans Criteria (NOC)	National Emergency X-Radiography Utilization Study (NEXUS)
GCS 13-15 AND witnessed loss of consciousness, amnesia, or confusion AND 1 of the following:	GCS 15 AND 1 of the following:	Any of the following:
High risk criteria:	- Headache	Evidence of skull fracture
- GCS < 15 at 2 hours after injury	- Vomiting	- Scalp hematoma
- Suspected skull fracture (open or depressed)	- Age > 60 years	- Neurologic deficit
- Any sign of basal skull fracture	- Drug/alcohol intoxication	- Abnormal level of alertness
- 2+ episodes of vomiting	- Persistent anterograde amnesia	- Abnormal behavior
- Age > 65 years	- Visible trauma above the clavicle	- Persistent vomiting
Medium risk criteria:	- Seizure	- Coagulopathy
- Amnesia before impact of 30+ minutes		- Age ≥ 65 years
Dangerous mechanism (pedestrian struck by a motor vehicle, an occupant ejected from a motor vehicle, or a fall from an elevation of 3 or more feet or 5 stairs)		

The Canadian CT Head Rule (CCHR) included patients with GCS 13-15 and loss of consciousness, amnesia, or disorientation [13]. In developing this rule, CTH was used at the discretion of the emergency medicine physician, which occurred in only 67% of patients. Since not all patients received CTH, it is unknown whether unscanned patients harbored “clinically important” but unrecognized lesions, though follow-up did not reveal neurologic decline in patients not receiving CTH.

The National Emergency X-Radiography Utilization Study (NEXUS-II) enrolled 13,728 patients who received CTH in 21 North American emergency rooms [24]. The authors were more inclusive in their definition of mild TBI, not requiring loss of consciousness or post-traumatic amnesia, and they used a more stringent definition of clinically important intracranial injury, e.g. contusion diameter >1.0 cm, subdural hematoma thickness >1.0cm. Only 12.8% of patients had none of the risk factors and were considered low risk. Additional published tools include the World Federation of Neurosurgical Societies criteria5and CT in Head Injury Patients [3]. 

Validation of clinical decision tools

Multiple studies have compared the above published clinical decision tools to assess validity. NOC and CCHR have been assessed most frequently, with most studies finding that NOC is more sensitive but less specific for detecting intracranial hemorrhage on CTH. With one exception [25], these decision rules have lacked 100% sensitivity, i.e. some patients with positive CTH would be missed. 

A group of four institutions in the Netherlands prospectively compared NOC with CCHR in 3,181 mild TBI patients using partially adapted criteria to better fit the patient population [2]. The authors found 99.2% and 87.2% sensitivity of NOC and CCHR, respectively, with specificities of 3.1% and 39.3%. Nine Canadian emergency departments prospectively studied the NOC against their own CCHR in a follow-up study [4]. As in their previous work, they defined mild TBI as patients with GCS 13-15 with loss of consciousness, amnesia, or disorientation. The sensitivities of CCHR and NOC were 93.1% and 98.6%, respectively, and specificities were 51.4% and 12.9%. 

Additional studies over four continents comparing these tools found sensitivities of 86-100% for NOC and 78-100% for CCHR, along with specificities of 10%-33% for NOC and 36-65% for CCHR [25,26]. Studies comparing NEXUS-II have demonstrated sensitivities of 85-99% and specificities of 26-47% [27]. These studies varied considerably in definitions of mild TBI and patient selection for CTH, but generally have established higher sensitivity and lower specificity with NOC compared to CCHR, with NEXUS-II falling in between the two. 

Current state

Debate continues over appropriate usage of CTH after mild TBI. While some have maintained that up to 35% of CTH ordered after mild TBI are unindicated based on prior observational studies [28], patterns of obtaining CTH have not demonstrably changed in the available literature. Our 8.5% positive CTH rate is consistent with the range of positive findings in prior studies, ranging from 6% to 12% depending on the definition of mild TBI and the country of study. 

Existing sets of criteria have demonstrated efficacy but have limitations, and the impact of these tools on clinical practice is questionable. In the best-studied example, additional education on CCHR at Canadian centers did not lessen rates of obtaining CTH [8]. In fact, the overall rate of obtaining CTH increased by about 10% over the three-year study period, and paradoxically the usage of CTH increased more in centers where the education took place. 

In our view, the difficulty in implementing decision tools into clinical practice is the concern for missing intracranial hemorrhages, whether or not the hemorrhage is clinically important. Anecdotal evidence suggests that some patients with small hemorrhages would be missed by any clinical decision rule, and this has likely driven what some perceive to be over-utilization of CTH. There may be a role for incorporation of observation period into decision rules, such as with CCHR and commonly-used pediatric decision rules [29], which may serve to decrease unnecessary radiation exposure without missing patients with hemorrhage. In the near future, serum biomarkers may also aid in identifying patients who do not need CTH.

We noted an older mean age among mild TBI patients with positive CTHs, a risk factor that has been identified in most prior analyses CTH [1,13,19]. We also found a high rate of positive CTH in male patients, which has not been noted previously. This finding is likely explained by other variables not available in this study, such as mechanism of injury and presenting signs and symptoms.

By using both trauma registry and non-trauma registry patients, our study highlights a limitation of using trauma registries for mild TBI studies. Many patients with mild head injury are not entered into our institution’s trauma registry, accounting for nearly half the cohort of patients presenting with GCS 13-15. The rate of traumatic intracranial hemorrhage was much lower in non-trauma registry patients (2.8% vs. 13.0%, odds ratio (OR) 5.1, p=0.0001), likely explaining why studies using National Trauma Data Bank (NTDB) have reported higher rates of positive CTH [30] than institutional series. 

Limitations

Limitations of this study include our inability to extrapolate clinical data from the radiologic database. While various keywords relating to mechanism of injury were used to identify scans for the study, these keywords alone were not useful in tabulating frequency of individual mechanisms, as many scans simply reported “trauma” as the clinical indication. Another drawback of our study design is that by querying CTHs obtained in the Emergency Department by specific words relating to blunt head trauma mechanism, a small percentage of patients may have been missed. If the indication for CT scan was very specific, the words we searched may not have been present within the report text. 

CTHs were obtained at the discretion of the Emergency Department, and only patients who received CTH were studied. Since we did not capture all mild traumatic brain patients, any patients with missed hemorrhages would not be included in our study, unless they returned to the Emergency Department and were scanned at that time. Prior work has generally presupposed that all intracranial hemorrhages after mild TBI are important, while anecdotal evidence suggests that some hemorrhages may not be clinically relevant. Additional study of mild TBI patient outcomes is warranted to identify which hemorrhages require intervention or close monitoring. Future decision rules designed to identify clinically important hemorrhages rather than any hemorrhage may be pertinent, particularly in resource-constrained environments.

The databases used in this study lack the clinical data needed for validation of clinical decision tools, including headache, vomiting, loss of consciousness, amnesia, and other symptoms. For this reason, risk factors for positive findings were not examinable beyond patient age and gender. Given these known barriers, our scope in this work was limited to assessing the incidence of positive CTH in our head trauma population.

## Conclusions

In a large series of patients receiving CTH for blunt head trauma, the rate of acute hemorrhage was 8.5%. Our study demonstrated older age and male sex to be significantly associated with positive CTH in trauma patients. Further studies, particularly those incorporating serum biomarkers, are warranted to better stratify patients in need of CTH after mild brain trauma.

## References

[1] Haydel MJ, Preston CA, Mills TJ, Luber S, Blaudeau E, DeBlieux PM (2000). Indications for computed tomography in patients with minor head injury. N Engl J Med.

[2] Smits M, Dippel DW, de Haan GG (2005). External validation of the Canadian CT Head Rule and the New Orleans Criteria for CT scanning in patients with minor head injury. JAMA.

[3] Smits M, Dippel DW, Steyerberg EW (2007). Predicting intracranial traumatic findings on computed tomography in patients with minor head injury: the CHIP prediction rule. Ann Intern Med.

[4] Stiell IG, Clement CM, Rowe BH (2005). Comparison of the Canadian CT Head Rule and the New Orleans Criteria in patients with minor head injury. JAMA.

[5] Servadei F, Teasdale G, Merry G (2001). Defining acute mild head injury in adults: a proposal based on prognostic factors, diagnosis, and management. J Neurotrauma.

[6] Levin HS, Diaz-Arrastia RR (2015). Diagnosis, prognosis, and clinical management of mild traumatic brain injury. Lancet Neurol.

[7] Bellolio MF, Heien HC, Sangaralingham LR (2017). Increased computed tomography utilization in the emergency department and its association with hospital admission. West J Emerg Med.

[8] Stiell IG, Clement CM, Grimshaw JM (2010). A prospective cluster-randomized trial to implement the Canadian CT Head Rule in emergency departments. CMAJ.

[9] Smith-Bindman R, Lipson J, Marcus R (2009). Radiation dose associated with common computed tomography examinations and the associated lifetime attributable risk of cancer. Arch Intern Med.

[10] Smits M, Dippel DW, Nederkoorn PJ (2010). Minor head injury: CT-based strategies for management--a cost-effectiveness analysis. Radiology.

[11] Stein SC, Burnett MG, Glick HA (2006). Indications for CT scanning in mild traumatic brain injury: a cost-effectiveness study. J Trauma.

[12] Lindor RA, Boie ET, Campbell RL, Hess EP, Sadosty AT (2015). Failure to obtain computed tomography imaging in head trauma: a review of relevant case law. Acad Emerg Med.

[13] Stiell IG, Wells GA, Vandemheen K (2001). The Canadian CT Head Rule for patients with minor head injury. Lancet.

[14] Clifton GL, Grossman RG, Makela ME, Miner ME, Handel S, Sadhu V (1980). Neurological course and correlated computerized tomography findings after severe closed head injury. J Neurosurg.

[15] Masters SJ, McClean PM, Arcarese JS (1987). Skull x-ray examinations after head trauma. Recommendations by a multidisciplinary panel and validation study. N Engl J Med.

[16] Stein SC, Ross SE (1990). The value of computed tomographic scans in patients with low-risk head injuries. Neurosurgery.

[17] Shackford SR, Wald SL, Ross SE (1992). The clinical utility of computed tomographic scanning and neurologic examination in the management of patients with minor head injuries. J Trauma.

[18] Nagy KK, Joseph KT, Krosner SM (1999). The utility of head computed tomography after minimal head injury. J Trauma.

[19] Jeret JS, Mandell M, Anziska B (1993). Clinical predictors of abnormality disclosed by computed tomography after mild head trauma. Neurosurgery.

[20] Reinus WR, Wippold FJ, 2nd 2nd, Erickson KK (1993). Practical selection criteria for noncontrast cranial computed tomography in patients with head trauma. Ann Emerg Med.

[21] Miller EC, Holmes JF, Derlet RW (1997). Utilizing clinical factors to reduce head CT scan ordering for minor head trauma patients. J Emerg Med.

[22] Ibanez J, Arikan F, Pedraza S (2004). Reliability of clinical guidelines in the detection of patients at risk following mild head injury: results of a prospective study. J Neurosurg.

[23] Jagoda AS, Bazarian JJ, Bruns JJ Jr. (2008). Clinical policy: neuroimaging and decisionmaking in adult mild traumatic brain injury in the acute setting. Ann Emerg Med.

[24] Mower WR, Hoffman JR, Herbert M (2005). Developing a decision instrument to guide computed tomographic imaging of blunt head injury patients. J Trauma.

[25] Papa L, Stiell IG, Clement CM (2012). Performance of the Canadian CT Head Rule and the New Orleans Criteria for predicting any traumatic intracranial injury on computed tomography in a United States Level I trauma center. Acad Emerg Med.

[26] Bouida W, Marghli S, Souissi S (2013). Prediction value of the Canadian CT head rule and the New Orleans criteria for positive head CT scan and acute neurosurgical procedures in minor head trauma: a multicenter external validation study. Ann Emerg Med.

[27] Mower WR, Gupta M, Rodriguez R, Hendey GW (2017). Validation of the sensitivity of the National Emergency X-Radiography Utilization Study (NEXUS) Head computed tomographic (CT) decision instrument for selective imaging of blunt head injury patients: An observational study. PLoS Med.

[28] Melnick ER, Szlezak CM, Bentley SK, Dziura JD, Kotlyar S, Post LA (2012). CT overuse for mild traumatic brain injury. Jt Comm J Qual Patient Saf.

[29] Kuppermann N, Holmes JF, Dayan PS (2009). Identification of children at very low risk of clinically-important brain injuries after head trauma: a prospective cohort study. Lancet.

[30] Kisat M, Zafar SN, Latif A (2012). Predictors of positive head CT scan and neurosurgical procedures after minor head trauma. J Surg Res.

